# Network pharmacology and molecular docking analyses on Lianhua Qingwen capsule indicate Akt1 is a potential target to treat and prevent COVID‐19

**DOI:** 10.1111/cpr.12949

**Published:** 2020-11-03

**Authors:** Qi‐Dong Xia, Yang Xun, Jun‐Lin Lu, Yu‐Chao Lu, Yuan‐Yuan Yang, Peng Zhou, Jia Hu, Cong Li, Shao‐Gang Wang

**Affiliations:** ^1^ Department of Urology Tongji Hospital Tongji Medical College Huazhong University of Science and Technology Wuhan China

**Keywords:** Akt1, Chinese patent medicine, COVID‐19, Lianhua Qingwen capsule, molecular docking, network pharmacology

## Abstract

**Objectives:**

Coronavirus disease 2019 (COVID‐19) is rapidly spreading worldwide. Lianhua Qingwen capsule (LQC) has shown therapeutic effects in patients with COVID‐19. This study is aimed to discover its molecular mechanism and provide potential drug targets.

**Materials and Methods:**

An LQC target and COVID‐19–related gene set was established using the Traditional Chinese Medicine Systems Pharmacology database and seven disease‐gene databases. Gene ontology (GO), Kyoto Encyclopedia of Genes and Genomes (KEGG) enrichment analysis and protein‐protein interaction (PPI) network were performed to discover the potential mechanism. Molecular docking was performed to visualize the patterns of interactions between the effective molecule and targeted protein.

**Results:**

A gene set of 65 genes was generated. We then constructed a compound‐target network that contained 234 nodes of active compounds and 916 edges of compound‐target pairs. The GO and KEGG indicated that LQC can act by regulating immune response, apoptosis and virus infection. PPI network and subnetworks identified nine hub genes. The molecular docking was conducted on the most significant gene Akt1, which is involved in lung injury, lung fibrogenesis and virus infection. Six active compounds of LQC can enter the active pocket of Akt1, namely beta‐carotene, kaempferol, luteolin, naringenin, quercetin and wogonin, thereby exerting potential therapeutic effects in COVID‐19.

**Conclusions:**

The network pharmacological strategy integrates molecular docking to unravel the molecular mechanism of LQC. Akt1 is a promising drug target to reduce tissue damage and help eliminate virus infection.

## Graphical abstract

A Lianhua Qingwen capsule (LQC) target and COVID‐19 related gene set is established to construct compound‐target pharmacology network. Gene ontology and Kyoto Encyclopedia of Genes and Genomes analysis indicate the regulating effect of LQC on apoptosis, antivirus, immune defense, and inflammatory response. Protein‐protein interaction network and critical subnetworks are constructed to identify hub gene target. The most significant gene, Akt 1, is selected to perform molecular docking with active compounds of LQC. Six compounds are finally recognized as potential anti‐COVID‐19 agents.

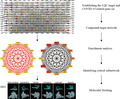

## 1 INTRODUCTION

Coronavirus disease 2019 (COVID‐19) is an acute respiratory infectious disease caused by severe acute respiratory syndrome coronavirus‐2 (SARS‐CoV‐2).^1^ It can lead to fever, fatigue, dry cough, multiple organ dysfunction and death.^2^ A total of 216 countries or regions have reported confirmed cases. By 4 September 2020, the number of patients has reached 26 171 112, including at least 1 million deaths.^3^ As a public health emergency with international concern, COVID‐19 has brought a disastrous impact on the global health system and economic system.^4^ However, the drug that can cure SARS‐CoV‐2 infection is still elusive.

China has successfully controlled the domestic epidemic in a short period due to the strict epidemic policy. In this process, traditional Chinese medicine (TCM) has also made a great contribution.^5^ A meta‐analysis that incorporated 11 studies compared TCM plus western medicine with western medicine alone.^6^ The pooled results showed that integrated TCM and western medicine generated a higher overall response rate, higher cure rate, lower severity illness rate and shorter hospital stay.^6^, ^7^ Besides, the Guideline on Diagnosis and Treatment of Coronavirus Disease 2019 (8th version) in China recommended various TCMs for patients in the medical observation period or different stages of infection, revealing favourable effects of TCM on symptom alleviation and reduction of severity conversion.^8^ Nevertheless, the mechanism by which TCM works is not clear since TCM usually consists of dozens of compounds for both Chinese patent medicine and Chinese herbal compound formulae. It is of clinical significance to explore the active compounds and target genes of TCM to guide drug discovery.

Among the diverse TCMs that can defend against SARS‐CoV‐2 infection and COVID‐19, Lianhua Qingwen capsule (LQC) shows great effectiveness in the treatment of patients with COVID‐19 both in clinical observation and randomized controlled trials (RCT)..^9^, ^10^, ^11^ LC is a Chinese patent medicine composed of 13 ingredients.^12^ LQC is widely used in preventing and treating viral influenza (eg, H1N1) in China.^12^ In the present SARS‐CoV‐2 pandemic, an RCT with 259 participants found that LQC plus abidor was associated with a higher overall response rate and comparable adverse events than abidor alone in mild cases.^11^ Another study developed a quadruple combination therapy including LQC and evaluated its efficacy.^10^ After treatment, coagulation disorder in severe COVID‐19 infection cases was significantly improved and patients in the combined therapy group had a better prognosis.^10^ The cumulative evidence proved the capability of LQC to control SARS‐CoV‐2 infection. Therefore, the study aims to identify the active components of LQC related to SARS‐CoV‐2 defence and investigate the key targets of eliminating the infection.

Akt is a serine/threonine protein kinase that includes Akt1, Akt2 and Akt3. Recent studies showed that during SARS‐CoV‐2 infection, Akt is activated in a dose‐dependent manner.^13^ The PI3K/Akt/mTOR pathway is also involved in lung injury,^14^ lung fibrogenesis^15^ and immune cell development.^16^ The results indicate that Akt may be a therapeutic target for COVID‐19. Network pharmacology is a novel method that integrates computer science and medicine, constructing and visualizing ‘multi‐gene, multi‐target, multi‐pathway’ interaction network to evaluate the molecular mechanism of drugs.^17^ This approach is perfectly suitable for the research of multi‐component drug such as TCM due to their complex matrices nature.^18^ Molecular docking refers to the process that a small molecular is spatially docked into a macromolecular and can score the complementary value at the binding sites, which is used for structure‐based drug design.^19^ In this study, we explored the molecular mechanism of the action of LQC in COVID‐19 using network pharmacology and molecular docking. We found that Akt1 was a hub gene that LQC primarily regulated, suggesting a novel target for COVID‐19 treatment.

## 2 MATERIALS AND METHODS

### 2.1 Obtaining the LQC target and COVID‐19–related gene set

First, we searched the main ingredients of Lianhua Qingwen capsule in the Traditional Chinese Medicine Systems Pharmacology (TCMSP) database (https://tcmspw.com/) to obtain the active compounds and their target genes.^20^ Specifically, we selected the “Herb name” by each ingredient of LQC, respectively. The search results showed a series of compounds in traditional Chinese medicine and their corresponding pharmacokinetic indicators. We filtered active compounds by setting the pharmacokinetic index that the oral bioavailability (OB) was greater than 30% and the drug‐like (DL) index was > 0.18. For each active compound, we searched related target genes in TCMSP. An LQC target gene set is acquired after gene symbol annotation under the help of Uniprot (https://www.uniprot.org/).^21^

Then, Seven databases were used to search COVID‐19–related genes: Genecards database (https://www.genecards.org/),^22^ OMIM database (https://omim.org/),^23^ PharmGkb database (https://www.pharmgkb.org/),^24^ TTD database (http://db.idrblab.net/ttd/),^25^ DrugBank database (https://www.drugbank.ca/),^26^ DisGeNet database (https://www.disgenet.org/home/)^27^ and PubChem database (https://pubchem.ncbi.nlm.nih.gov/).^28^ We established a COVID‐19–related gene set by taking a union of the search results.

An LQC target and COVID‐19–related gene set was obtained by intersecting the LQC target gene set and the COVID‐19–related gene set.

### 2.2 Compound‐target pharmacology network and enrichment analysis

Based on the LQC target gene set and the COVID‐19–related gene set, a compound‐target network is constructed by means of Cytoscape version 3.8.0.^29^ Enrichment analysis, including gene ontology (GO) and Kyoto Encyclopedia of Genes and Genomes (KEGG) pathway analysis, was performed to reveal the underlying mechanism through biological processes, cellular components, molecular function and key signalling pathways. The “clusterprofile” package in R software version 3.4.0 was used to performed enrichment analysis.

### 2.3 Protein‐protein interaction (PPI) network and critical subnetwork

The LQC target and COVID‐19–related gene set was used to construct PPI network by using STRING database.^30^ We set the parameter as moderate confidence (0.400). The PPI network from STRING was then imported into Cytoscape to investigate the critical subnetwork. We applied two methods to screen the core subnetwork. Firstly, we used CytoNca plugin in Cytoscape to analyse the PPI network.^31^ In detail, we filtered genes according to the primary score file calculated by CytoNca that each score of Betweenness, Closeness, Degree, Eigenvector, LAC, network scores was higher than the median value. We constructed a primary subnetwork using the filtered genes. The filter process was conducted again to acquire the final critical subnetwork. Another method we used to screen critical subnetwork was CytoHubba plugin in Cytoscape. This approach was to analyse the top 12 genes in the PPI network and to construct the critical subnetwork without checking the first‐stage nodes.

### 2.4 Molecular docking technology

The most significant gene from two critical subnetworks was selected for subsequent molecular docking analysis. The receptor protein coded by the selected gene was searched in the Uniprot database (https://www.uniprot.org/). We downloaded 3D structure of the protein in RCSB PDB database (https://www.rcsb.org/). The 2D structure for the molecule ligands was downloaded from the PubChem database (https://pubchem.ncbi.nlm.nih.gov/). ChemBio 3D software was used to calculate and export the 3D structure by minimizing energy. PyMOL 2.4.0 software was performed the dehydration of the receptor protein and Autodock software was used to carry out hydrogenation and charge calculation of proteins. Parameters of the receptor protein docking site were set to include the active pocket sites where small molecule ligands bind. Finally, Autodock Vina was used to dock the receptor protein with the small molecule ligands of the active compounds of LQC.^32^

## 3 RESULTS

### 3.1 Screening of active compounds and potential targets

**Figure 1:**
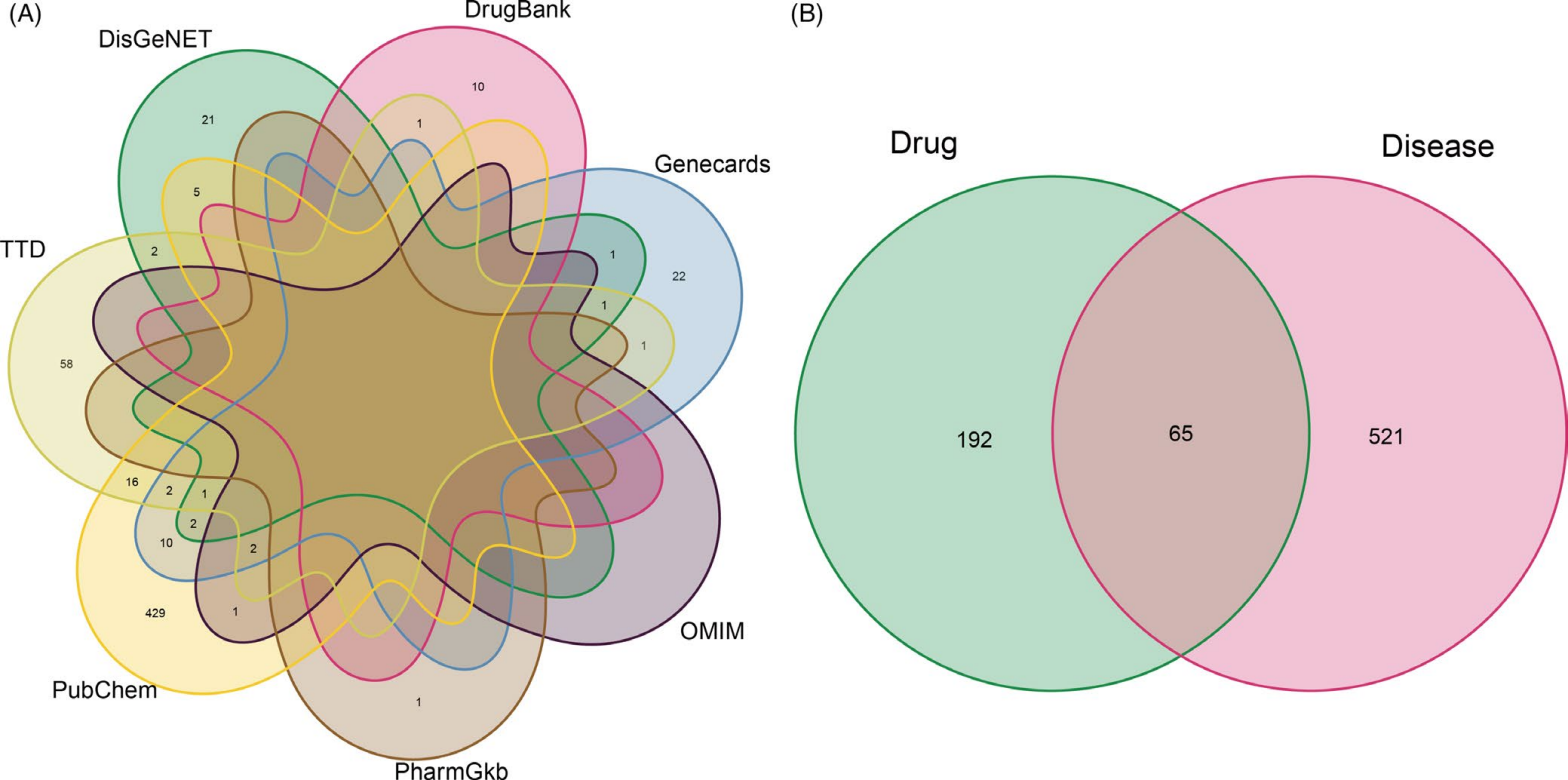
Identification of the drug‐target interaction. A, Identification of the COVID‐19–related genes by taking a union of all the results from 7 database. B, Identification of the drug‐target disease‐related genes by taking an intersection of drug target genes and COVID‐19–related genes

Using the TCMSP database, 10 key ingredients of LQC were obtained: Banlangen, Dahuang, Gancao, Guanghuoxiang, Guanzhong, Jinyinhua, Kuxingren, Lianqiao, Yuxingcao and Gancao. A total of 257 drug target genes were gained. Besides, we obtained 42, 5, 1, 87, 11, 33 and 473 COVID‐19–related genes from Genecards, OMIM, PharmGkb, TTD, DrugBank, DisGeNet and PubChem database, respectively. After removing duplication and combining the search results, an overall 586 COVID‐19–related gene set was acquired (Figure 1A). Further, by taking an intersection of the compound‐target genes and disease‐related genes, we finally obtained the LQC target and COVID‐19–related gene set (Figure 1B).

### 3.2 Compound‐target network

**Figure 2:**
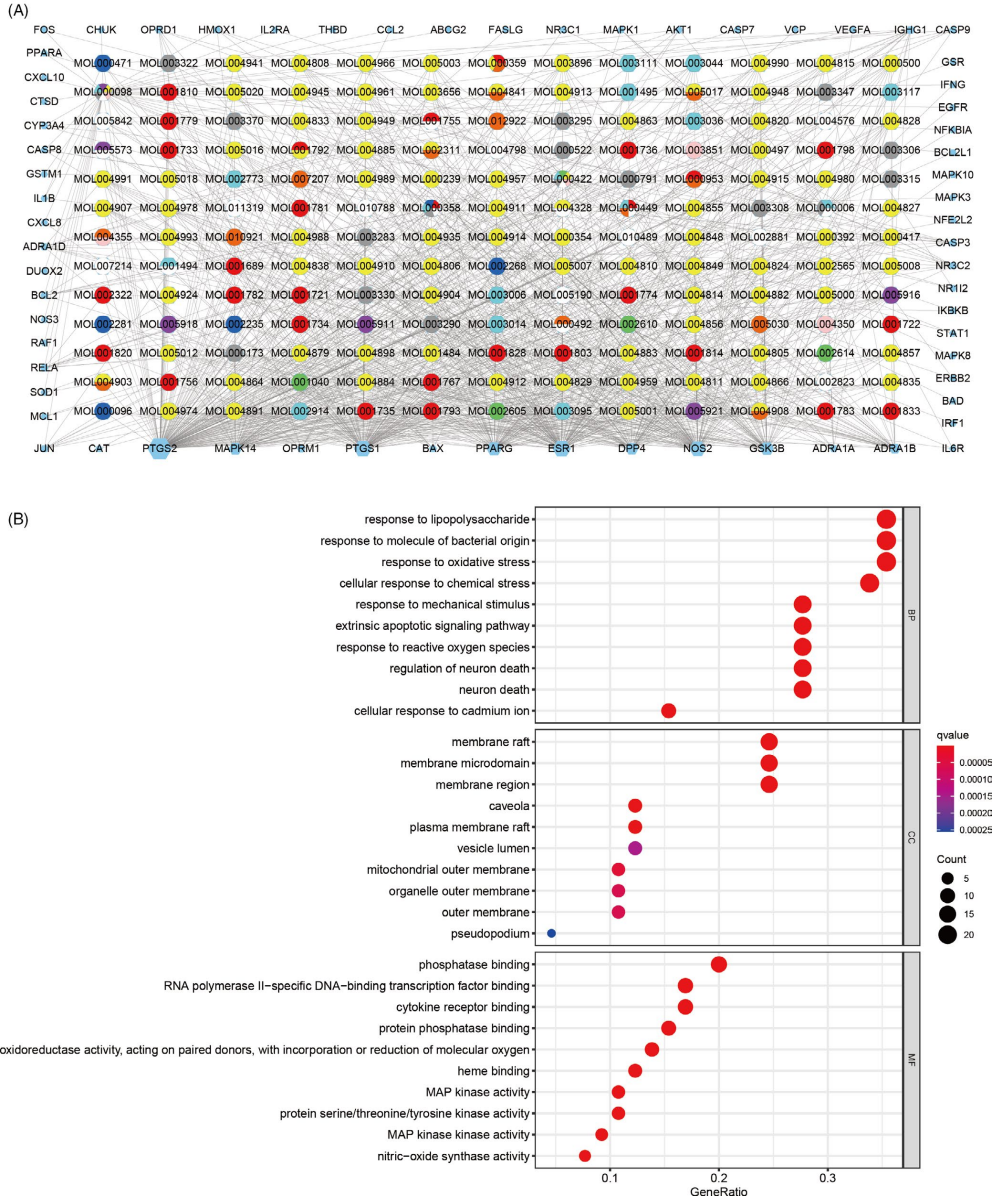
Construction of the drug‐target pharmacology network and GO enrichment analysis. A, The drug‐targets interaction pharmacology network. Circles represent the small molecule active compounds in LQC. Each colour represents a traditional Chinese medicine ingredient. Hexagon represents the COVID‐19–related target genes, and edges represent the interaction between the small molecule compounds and the target genes. B, GO enrichment analysis of the target genes. Gene ratio refers to the ratio of enriched genes to all target genes, and counts refer to the number of the enriched genes

After discovering compound‐target disease‐related genes, we visualized the compound‐target interaction network with 234 nodes and 916 edges by using Cytoscape 3.8.0 (Figure 2A). Generally, one gene is targeted by multiple active compounds while one compound can target more than one gene. Among 65 genes, PTGS 2 is the most targeted gene by LQC ingredients.

### 3.3 GO enrichment analysis

GO enrichment analysis was used to discover the underlying biological processes (BP), cellular components (CC) and molecular functions (MF) of the 65 target genes. By setting the filter as adjusted *P*‐value <0.05 and *q*‐value < 0.05, we obtained 1711 significant enriched GO terms. The top 10 terms were illustrated in Figure 2B. The GO terms suggested that these target genes played an essential role in host defence and response to stress. Additionally, we exhibited 7 GO terms related to virus invasion from the enrichment analysis results as Table 1, which suggested that these target genes play a significant role in virus infection.

**Table 1:** Virus‐related GO terms enriched by the target genes

Ontology	ID	Description	Gene ratio	*P*‐value	*P*‐adjust	*q*‐value	Count
BP	GO:0009615	Response to virus	12/65	1.04E‐09	3.60E‐08	1.47E‐08	12
BP	GO:0051607	Defence response to virus	8/65	1.67E‐06	1.96E‐05	8.00E‐06	8
BP	GO:0019054	Modulation by virus of host cellular process	3/65	3.17E‐05	.000222	9.07E‐05	3
BP	GO:0019048	Modulation by virus of host process	3/65	.000169	.000887	0.000362	3
BP	GO:0050688	Regulation of defence response to virus	3/65	.002105	.006533	0.00267	3
BP	GO:0050691	Regulation of defence response to virus by host	2/65	.007349	.017575	0.007182	2
BP	GO:0098586	Cellular response to virus	2/65	.014672	.030848	0.012607	2

### 3.4 KEGG enrichment analysis

**Figure 3:**
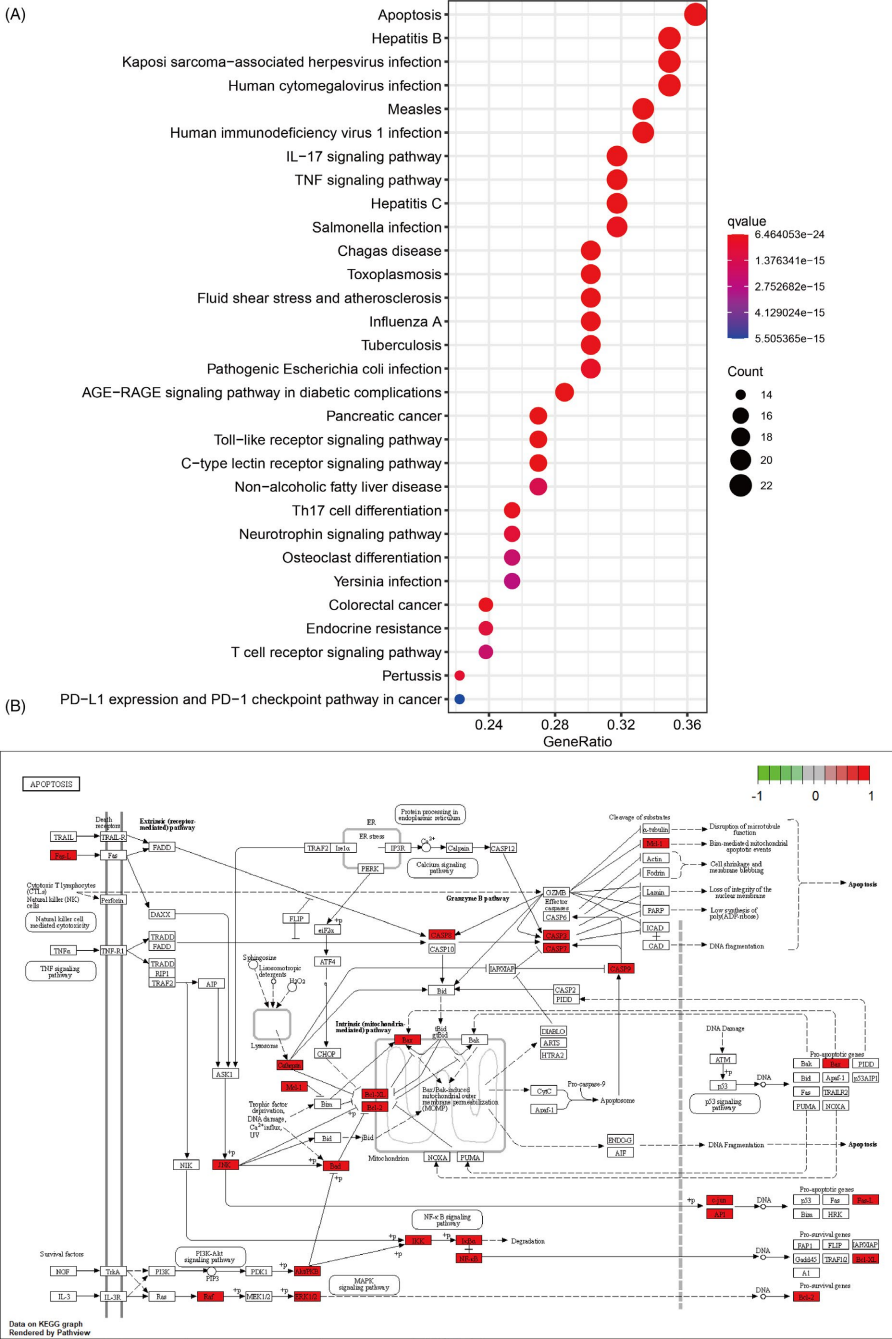
KEGG enrichment analysis and pathway map. A, KEGG enrichment analysis of the target enes. Gene ratio refers to the ratio of enriched genes to all target genes. Counts refer to the number of the enriched genes. B, Pathway map of apoptosis as the most significant enriched pathway

KEGG enrichment analysis was performed to discover those pathways enriched by the 65 target genes. The filter was also set as an adjusted *P*‐value <0.05 and *q*‐value < 0.05. A total of 151 KEGG pathways were significantly enriched, which showed that these target genes affected the pathways of bacterial and viral infection, the differentiation of immune cells and signal transduction pathways, as well as a series of important pathological processes such as apoptosis. The bubble plot of the most significant 30 KEGG pathways was shown in Figure 3A and the pathway map of the apoptosis was illustrated in Figure 3B. In addition, we extracted and exported the virus‐related pathways as Table 2, and the pathway map can be acquired in the supplementary file.

**Table 2:** Virus‐related pathway enriched by target genes

ID	Description	Gene ratio	*P*‐value	*P*‐adjust	*q*‐value	Count
hsa05161	Hepatitis B	22/63	2.84E‐22	1.19E‐20	2.92E‐21	22
hsa05167	Kaposi sarcoma‐associated herpesvirus infection	22/63	1.47E‐20	3.24E‐19	7.98E‐20	22
hsa05160	Hepatitis C	20/63	1.18E‐19	2.16E‐18	5.33E‐19	20
hsa05163	Human cytomegalovirus infection	22/63	4.39E‐19	6.79E‐18	1.67E‐18	22
hsa05170	Human immunodeficiency virus 1 infection	21/63	2.54E‐18	3.20E‐17	7.87E‐18	21
hsa05164	Influenza A	19/63	1.59E‐17	1.89E‐16	4.64E‐17	19
hsa05169	Epstein‐Barr virus infection	16/63	1.73E‐12	8.08E‐12	1.99E‐12	16
hsa05165	Human papillomavirus infection	18/63	3.33E‐11	1.29E‐10	3.17E‐11	18
hsa05166	Human T‐cell leukaemia virus 1 infection	14/63	9.05E‐10	3.03E‐09	7.46E‐10	14
hsa05203	Viral carcinogenesis	9/63	2.58E‐05	5.29E‐05	1.30E‐05	9
hsa04061	Viral protein interaction with cytokine and cytokine receptor	5/63	.001056	.001768	0.000435	5
hsa05416	Viral myocarditis	3/63	.011338	.016395	0.004035	3

### 3.5 PPI network and core subnetwork

**Figure 4:**
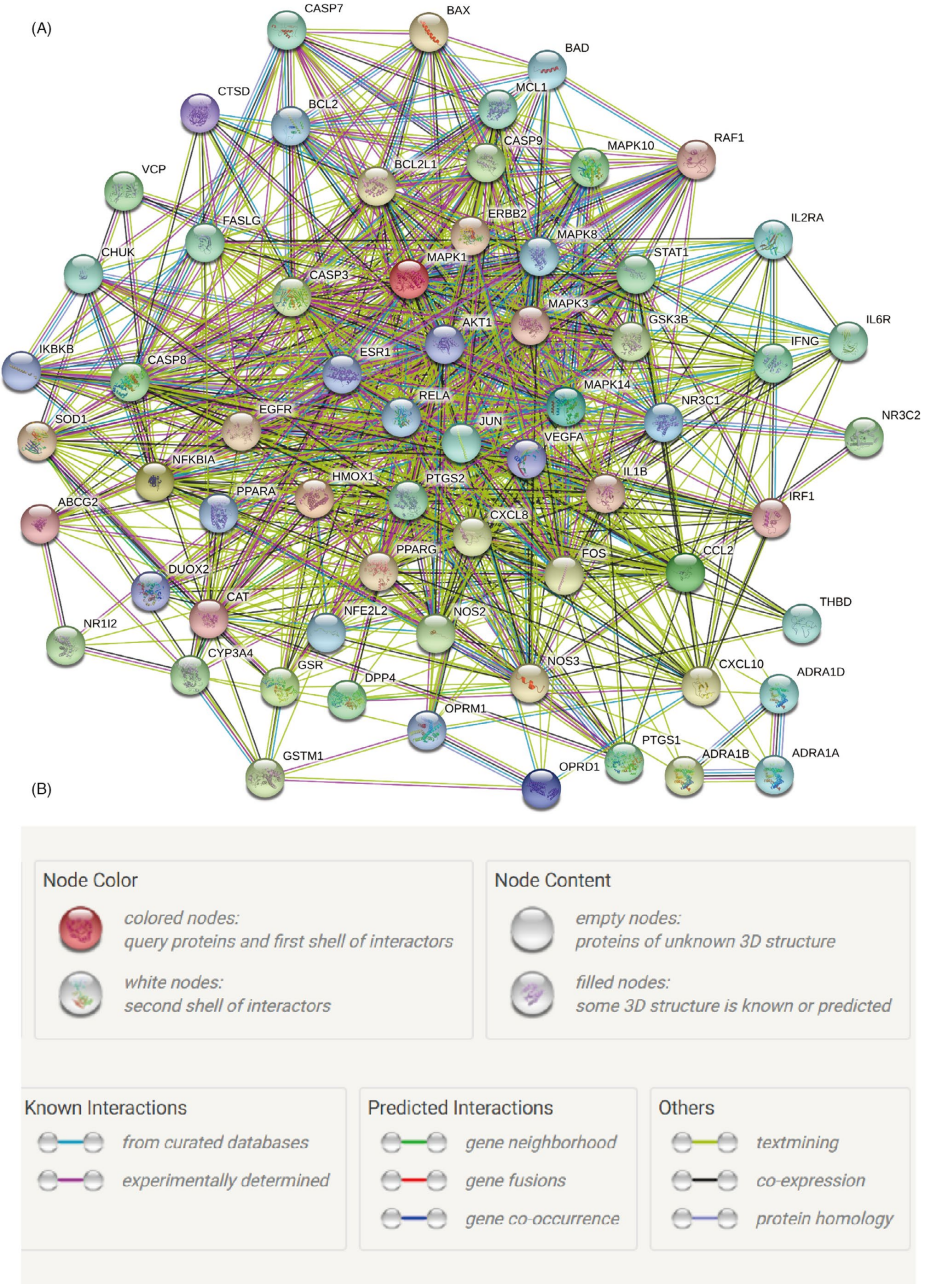
Protein‐Protein interaction (PPI) network. A, PPI network exported from STRING database. B, Annotations for the nodes and edges in the PPI network

**Figure 5:**
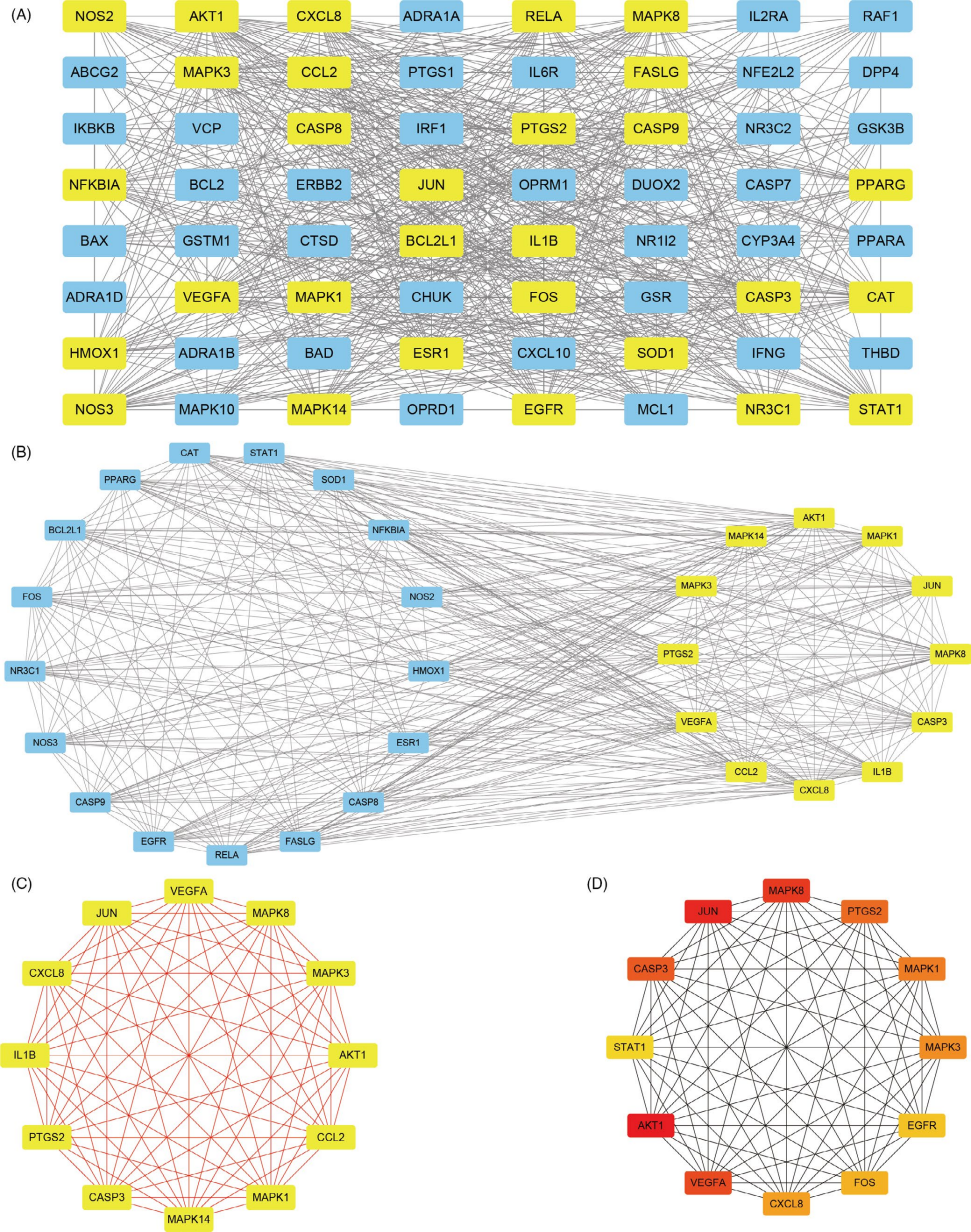
Identification of key subnetwork using Cytoscape. A, PPI network and the first filtration by CytoNca, the yellow nodes were screened with each score higher than median. B, Subnetwork constructed by a second filtration via CytoNca. The yellow nodes were screened with a score higher than the median. C, Final key subnetwork screened after two filtrations using CytoNca. D, Key subnetwork of top 12 nodes analysed by CytoHubba

Protein‐protein interaction network derived from STRING database showed that the proteins encoded by these target genes had complex interactions (Figure 4A, B). We imported PPI network into Cytoscape for further analysis. Finally, two key subnetworks composed of 12 target genes were obtained by using CytoNca and CytoHubba, respectively (Figure 5A‐D).

### 3.6 Molecular docking of active compounds and Akt1 encoding protein

**Figure 6:**
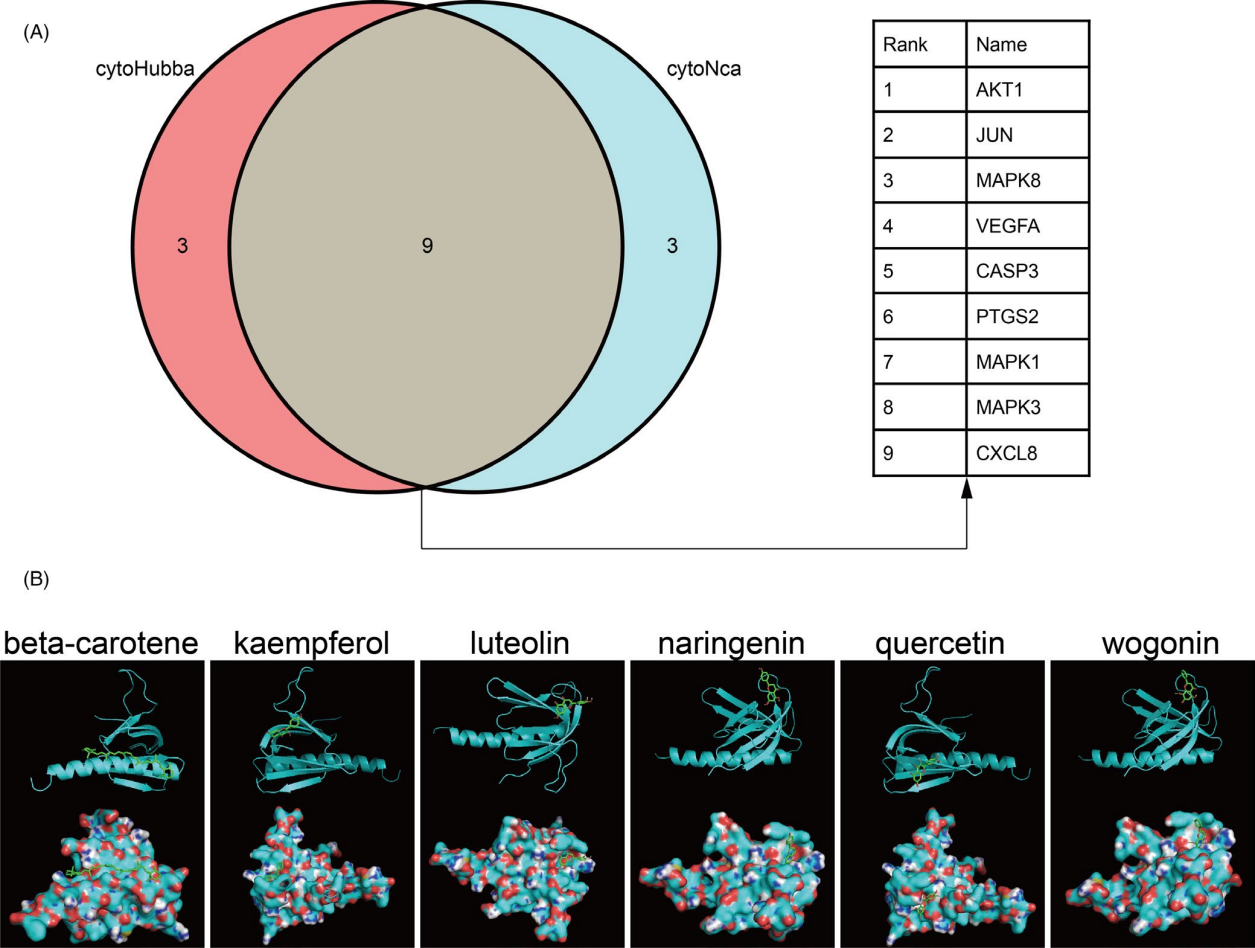
Screening of the key genes in the subnetwork and further molecular docking. A, Screening of the key genes by taking an intersection of the two key subnetworks. B, Molecular docking between the six small molecule ligands and protein 1UNP (encoded by AKT1), on the top shows the 3D structure of ligands and receptors, at the bottom shows the surface of the receptor and 3D structure of the ligands

We took an intersection of the two key subnetworks (Figure 6A) and nine genes with their rank of significance. The most significant gene, Akt1, was selected to conduct molecular docking. We then obtained six active compounds targeting Akt1 protein from the compound‐target interaction network. The compounds were beta‐carotene, kaempferol, luteolin, naringenin, quercetin and wogonin. Subsequently, molecular docking indicated that all these six active compounds could easily enter and bind the active pocket of the Akt1 protein as shown in Figure 6B. The docking scores were recorded in Table 3.

**Table 3:** Molecular docking score

Molecule name	Docking score (kcal/mol)
Beta‐carotene	‒6.5
Kaempferol	‒6.8
Luteolin	‒7.3
Naringenin	‒7.6
Quercetin	‒7.0
Wogonin	‒7.8

## 4 DISCUSSION

Over the past ten months, COVID‐19 has rapidly spread around the world. SARS‐CoV‐2 pandemic is still raging in most countries due to the lack of target drugs. Notably, China, as a country with a population of more than 1.3 billion, has successfully controlled the epidemic outbreak. TCM has made an indispensable contribution to prevent and cure SARS‐CoV‐2 infection. Among all the anti‐COVID‐19 TCMs, LQC is the main Chinese patent medicine that is recommended by the Guideline on Diagnosis and Treatment of Coronavirus Disease.^8^ Studies have confirmed the efficacy of LQC in symptom relief and clinical outcome improvement of patients with COVID‐19.^6^, ^9^, ^10^, ^11^ In the present study, we constructed an LQC target COVID‐19–related gene set that consisted of 65 target genes by analysing the active components from 10 ingredients of LQC. The compound‐target network depicted the compound‐target pairs. GO and KEGG analysis revealed that LQC can regulate the process of immune pathways and virus defence. PPI network and critical network analyses found 9 hub targets out of 65 genes. We focus on the most significant gene, Akt1, and performed molecular docking to verify the interaction between active compounds of LQC and Akt1. The results of the research demonstrate the effectiveness of LQC in the treatment of COVID‐19 from a bioinformatics perspective, and provide a landscape on the mechanism of LQC. The results may also promote target drug design and basic research on SARS‐CoV‐2 infection.

We screened several ingredients of LQC in TCMSP database. Banlangen, one of the most important ingredients of LQC, can act on various viruses such as HCMV, influenza virus, HBsAg and HBV‐DNA, which is consistent with KEGG analysis in our study.^33^ It can also combat oxygen free radicals through reducing the synthesis and secretion of inflammatory mediators like TNF‐α, IL‐6 and IL‐10 that are considered as critical markers for disease severity and poor prognosis of COVID‐19.^34^, ^35^ Furthermore, erucic acid isolated from Banlangen can suppress alveolar epithelial apoptosis initiated by influenza A virus via NF‐κB and p38 MAPK pathway, indicating a therapeutic potential for T‐cell apoptosis tendency triggered by SARS‐CoV‐2.^36^, ^37^, ^38^ Guanghuoxiang, also named pogostemon cablin, is a well‐known Chinese *materia medica*. Kiyohara et al exhibited a 99.8% inhibitory effect against the H1N1 influenza virus at a concentration of 10 μg/mL methanol extract from Guanghuoxiang.^39^ Another effective ingredient Jinyinhua is used in multiple Chinese patent medicine. Ethanol extract from the herb can substantially decrease the release of nitric oxide, IL‐6 and TNF‐α in macrophage. A large number of mechanistic studies of these ingredients can predict the efficacy of LQC in preventing SARS‐CoV‐2 infection, involving antiviral, anti‐inflammation and anti‐apoptosis effects.

The results of the target genes enrichment analysis by GO and KEGG are interesting. Firstly, target genes were found enriched in the defence and regulation of viral infection, which might directly influence the results of viral infection. This result is consistent with previous studies. Yang et al showed that LQC displayed antiviral and anti‐inflammatory activity and synergistic effects with oseltamivir against influenza B virus infection.^40^ Our study further demonstrated the feasibility of LQC in the treatment of COVID‐19. Moreover, the 10 most significant GO (BP) terms indicated that LQC could regulate the oxidative stress process during the treatment of COVID‐19. Schönrich et.al reported that the overwhelming production of reactive oxygen species resulting in oxidative stress is a major cause of local or systemic tissue damage that leads to severe COVID‐19.^41^ It has been reported that some surface proteins in SARS‐CoV‐2 can bind to the haemoglobin molecule of an erythrocyte, resulting in the destruction of the haem structure and the release of harmful iron ions into the blood, which lead to the development of oxidative stress and bring oxidative damage to the tissues and organs.^42^ From this point of view, we speculated that LQC may treat COVID‐19 by antagonizing oxidative stress damage and injury. This oxidative stress‐based concept of COVID‐19 pathogenesis and treatment should be validated in randomized controlled clinical studies and deeper molecular studies. Moreover, target genes were also enriched in the MAPK and MAPKK activation pathways. Several studies have demonstrated that the MAPK pathway is closely related to SARS‐CoV. Lee et al^43^ found that phosphorylated p38 MAPK was increased in CD14‐positive monocytes in SARS patients. Augmented p38 MAPK activation in CD14 cells is associated with elevated IL‐8 levels.^43^ Moreover, the p38 MAPK signalling pathway is also implicated in the death of SARS‐COV–infected cells.^44^ Recently, Zhang et al reported that SARS‐CoV‐2–induced platelet activation may participate in thrombus formation and inflammatory responses in COVID‐19 patients.^45^ MAPK pathway, located downstream of ACE2, mediated the potentiating role of SARS‐CoV‐2 on platelet activation, and that platelet ACE2 expression decreases following SARS‐COV‐2 stimulation.^45^ Our study indirectly shows that the MAPK pathway may play an important role in the treatment of COVID‐19 with LQC.

We mainly focus on the Akt1 gene as one of the critical nodes in the subnetworks and performed molecular docking between micromolecules and the coded protein. Akt1 is one of the serine/threonine protein kinases call Akt kinase (Akt1, Akt2 and Akt3).^46^ A previous study has shown that overexpressed constitutively active Akt1 can promote viral protein synthesis.^47^ Also, activation of the PI3K/Akt pathway is indispensable for coxsackievirus B3 infection.^48^ Dominant negative mutant of Akt1 can significantly dampen viral RNA expression and further reduce viral capsid protein expression and viral release. ^48^ The replication of another coronavirus, Middle East respiratory syndrome coronavirus, can be remarkably inhibited by administrating kinase inhibitors targeting the PI3K/Akt.^49^ Collectively, Akt1 could be an ideal target with a broad‐spectrum antiviral effect. After molecular docking, six molecules were found to directly interact with Akt1: beta‐carotene, kaempferol, luteolin, naringenin, quercetin and wogonin. Among them, kaempferol has proven its protective effect against H9N2 swine influenza virus infection.^50^ Quercetin is also a potent antiviral agent against the influenza virus and coronavirus.^51^, ^52^, ^53^ Further studies are expected to evaluate the synergistic effect of these molecules.

The activation of PI3K/Akt/mTOR pathway is involved in pulmonary fibrosis and lung injury by regulating lung fibroblasts and lung epithelial cells. Transforming growth factor‐β (TGF‐β) is a common agent to induce the differentiation of fibroblasts into myofibroblasts, accompanied by the excessive secretion of extracellular matrix.^15^ In this process, the PI3K/Akt/mTOR pathway is upregulated by TGF‐β to increase the expression of the enzymes that are required for the deposition of collagen proteins and progressive scarring.^54^ Drugs such as isoliquiritigenin and Yifei Sanjie formula can improve TGF‐β induced pulmonary fibrosis through decreasing the phosphorylation levels of PI3K, Akt and mTOR.^55^, ^56^ Chronic radon exposure can cause lung injury and fibrosis, manifested as increasing lung epithelial cell proliferation and migration.^14^ Radon radiation also facilitates the phosphorylation of PI3K, Akt and mTOR.^14^ Fine particulate matter (PM2.5) is a primary air pollutant to cause lung injury. A mouse model study showed that PM2.5 can suppress bronchial epithelial cell autophagy by activating the PI3K/Akt/mTOR pathway.^57^ Lipopolysaccharide (LPS) can activate the TLR4/PI3K/Akt/mTOR pathway and further leads to neutrophil infiltration and alveolar wall oedema involving in acute lung injury.^58^ All the evidence indicates that targeting PI3K/Akt/mTOR pathway can protect lung epithelial cells and reduce fibrogenesis.

Akt also plays an essential role in immune cell modulation. Akt can regulate the development and functions of innate immune cells, such as neutrophil, macrophage and dendritic cell.^59^ The activation of Akt pathway aggregates inflammatory and metabolic signals, which regulates macrophage responses modulating their activation phenotype.^60^ In addition, Akt signalling is crucial in cellular immune response. In the course of acute infection, T cells expand and differentiate into effector cells, which mediate the destruction of the infected cells. Akt can be used as a key signalling node in the development of protective memory CD8 + T‐cell responses.^16^ Beyond that, Akt can also mediate the early metabolic response of naive human CD4 + T cell to TCR stimulation.^61^ Moreover, Akt is closely related to B cell. During the germinal centre response, The Akt isoforms 1 and 2 and its downstream pathways drive B cell fate decisions.^62^ Li et al^63^ reported that the Akt‐dependent inactivation of GSK3 and TSC1/2 can regulate B cell growth and metabolism in the B cell‐mediated immunity. In other words, Akt signalling plays a critical role in immune cell differentiation, proliferation and migration, which involved in the formation of systemic and local inflammation. However, hyperactivation of Akt during virus infection can lead to an elevation of terminal differentiated effector CD8 T cells and a subsequent elevation of senescent CD8 T cells.^64^ The immune cascade may rapidly cause T‐cell exhaustion and significantly increase the risk of death of patients infected with SARS‐CoV‐2.^65^ Inhibition of the overactivation of Akt during COVID‐19 may modulate immune response and improve prognosis.

In this pharmacology network–based study, we investigated the potential therapeutic mechanisms of the Chinese medicine LQC in COVID‐19. The results highlight the improvement in inflammatory response, cell apoptosis and immune defence of LQC antagonizing SARS‐CoV‐2 infection. Additionally, we provide several potential targets for COVID‐19 treatment, which could contribute to the development of new therapeutic strategies. However, the in‐depth mechanism of these active compounds still requires further elucidation, which may guide the design of novel broad‐spectrum antiviral agents.

## 5 CONCLUSION

We uncovered the potential mechanisms of LQC by employing pharmacology network and molecular docking computational analyses. We believe these findings may aid the global fight against the COVID‐19 pandemic.

## CONFLICT OF INTEREST

The authors declare no conflict of interest.

## AUTHOR CONTRIBUTIONS

Conception and design: Qi‐Dong Xia, Jia Hu, Cong Li, and Shao‐Gang Wang. Acquisition of data: Qi‐Dong Xia and Yu‐Chao Lu. Analysis of data: Qi‐Dong Xia, Yang Xun, Jun‐Lin Lu, and Yuan‐Yuan Yang. Interpretation of data: Yang Xun, Jun‐Lin Lu, and Peng Zhou. Drafting the manuscript: Qi‐Dong Xia, Yang Xun, and Jun‐Lin Lu. Revising the manuscript: All authors. All authors have approved the final version to be published, and agree to be responsible for all aspects of the work.

## Supporting information

Supplementary Material

## Data Availability

Source data of this study is derived from the public repositories, as indicated in the section of ‘Materials and Methods’ of the manuscript. All data that support the findings of this study is available from the corresponding author upon reasonable request.
