# Supplementary material for: Network pharmacology and molecular docking analyses on Lianhua Qingwen capsule indicate Akt1 is a potential target to treat and prevent COVID‐19
Source: Cell Prolif. 2020 Nov 3;53(12):e12949. doi: 10.1111/cpr.12949 (PMC7705900; doi:10.1111/cpr.12949)

**Supplementary file. Pathway map of virus related pathway enriched by target genes**

hsa05161. Hepatitis B


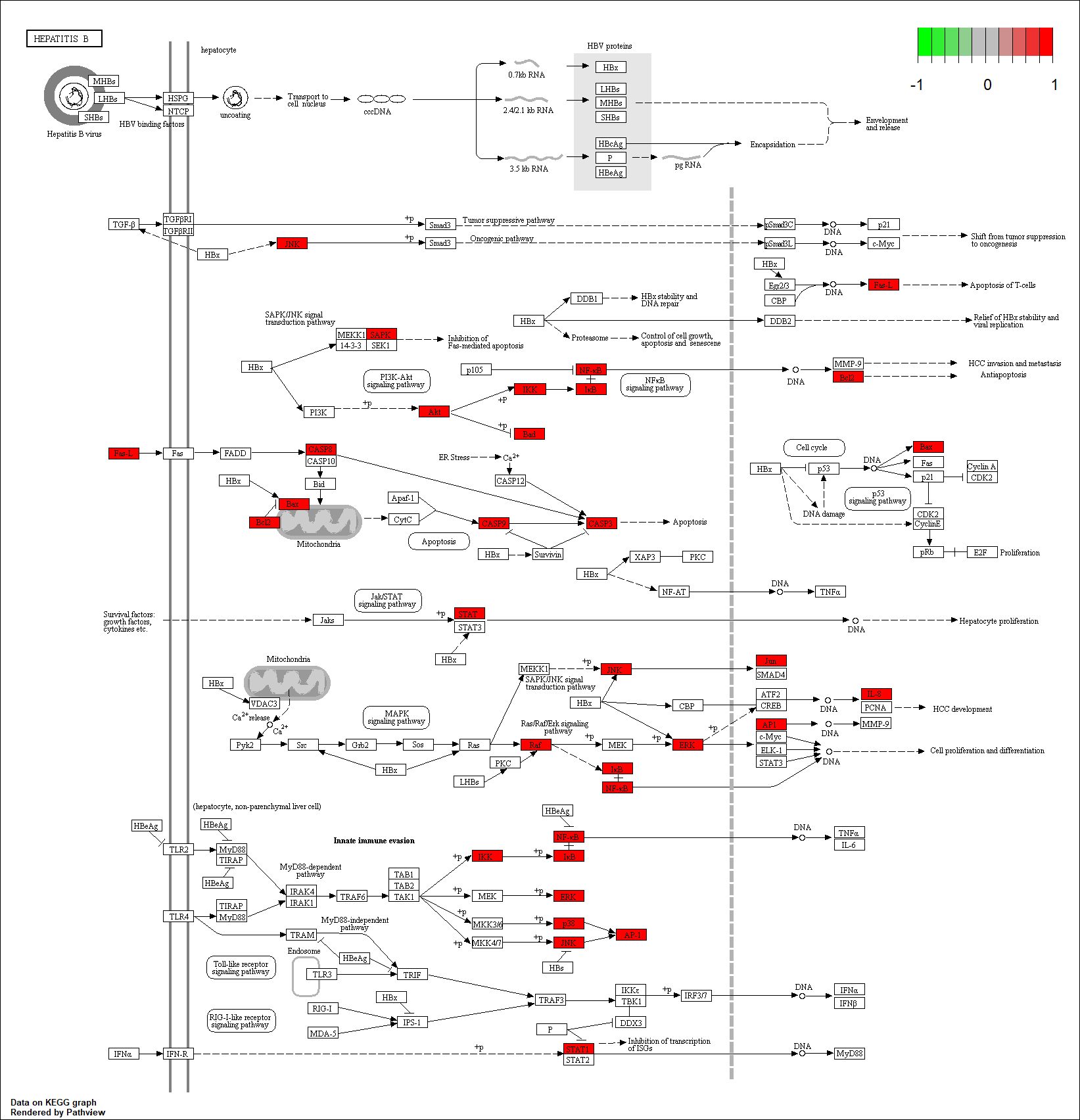


hsa05167. Kaposi sarcoma-associated herpesvirus infection


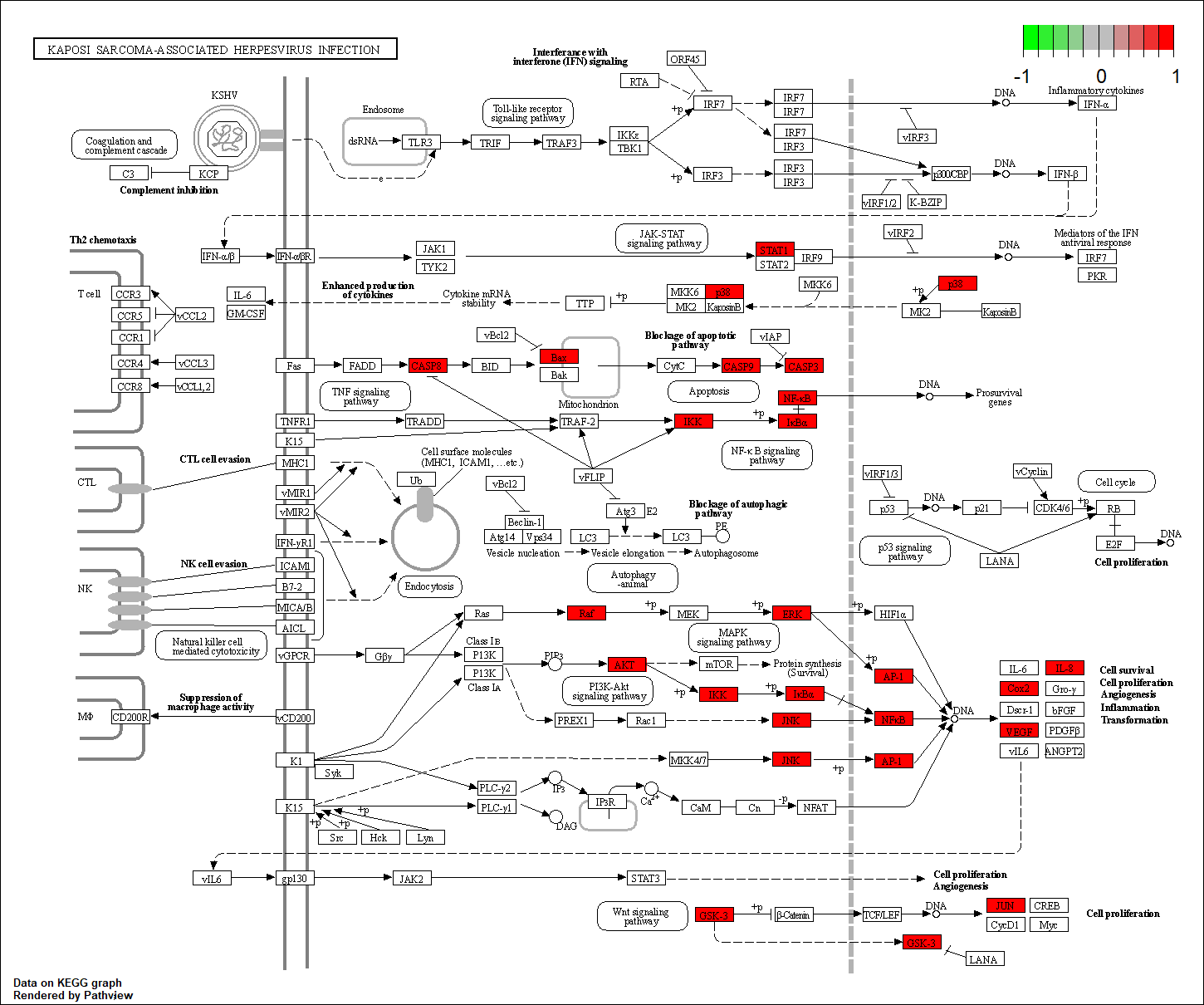


hsa05160. Hepatitis C


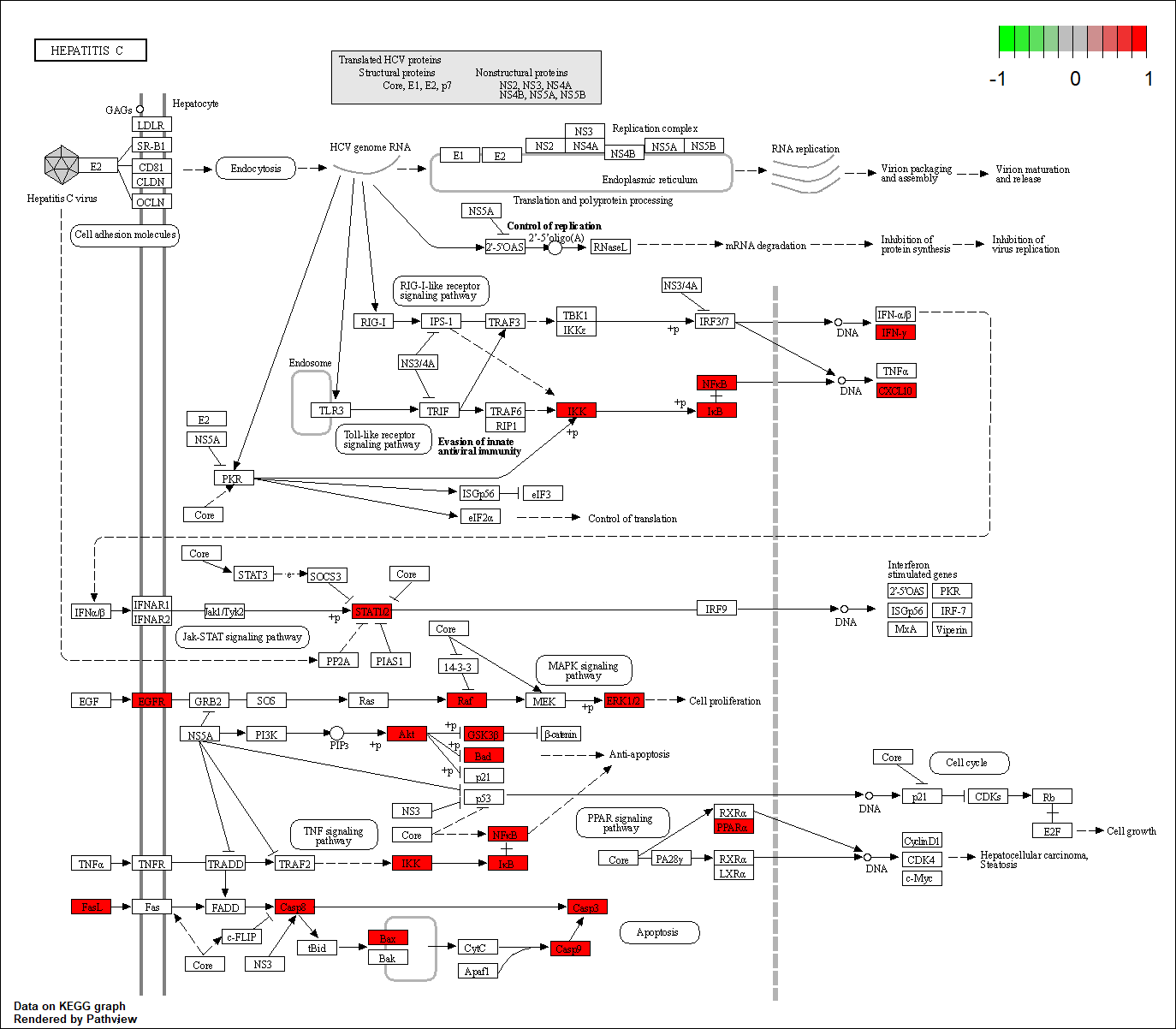


hsa05163. Human cytomegalovirus infection


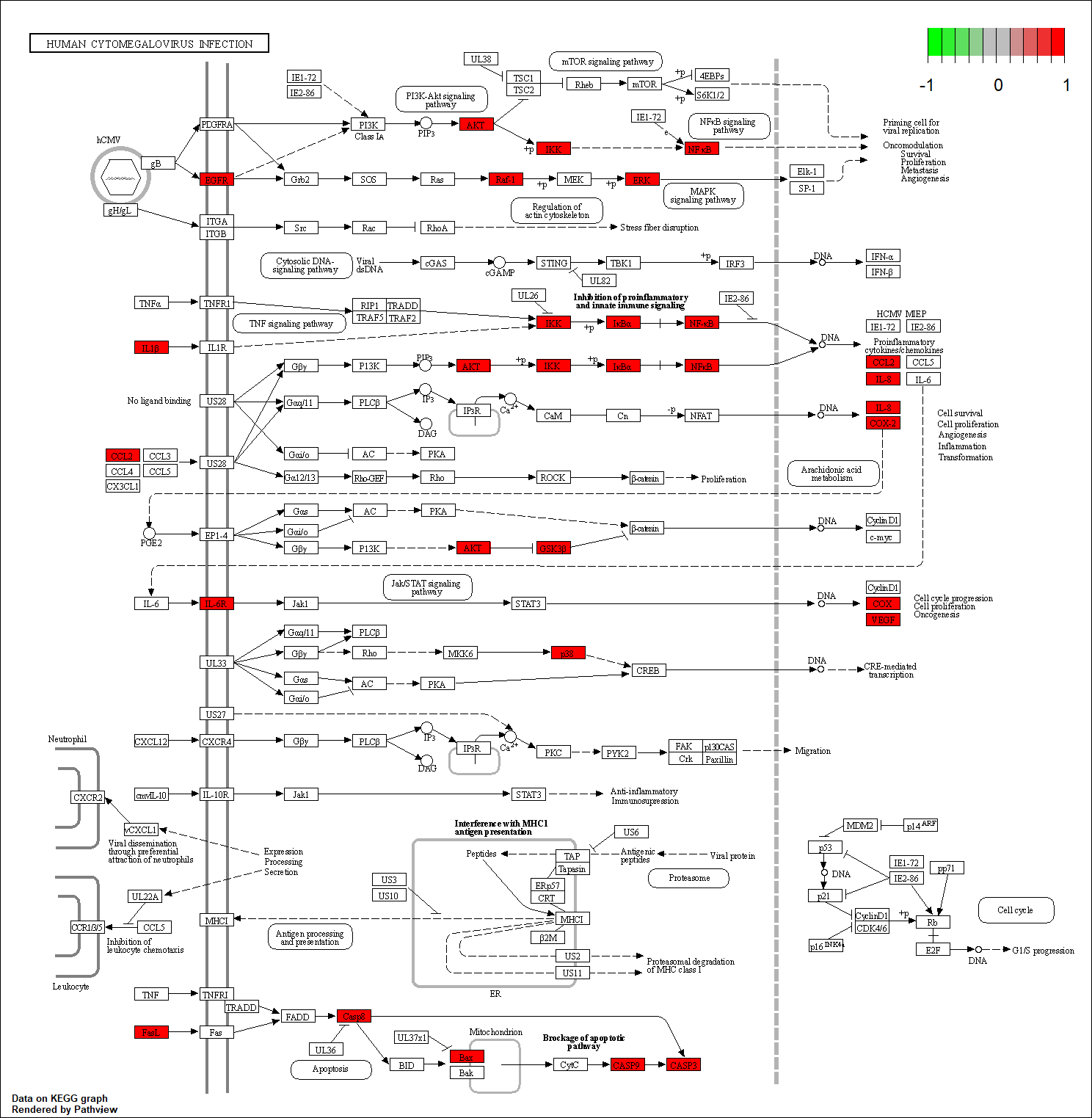


hsa05170. Human immunodeficiency virus 1 infection


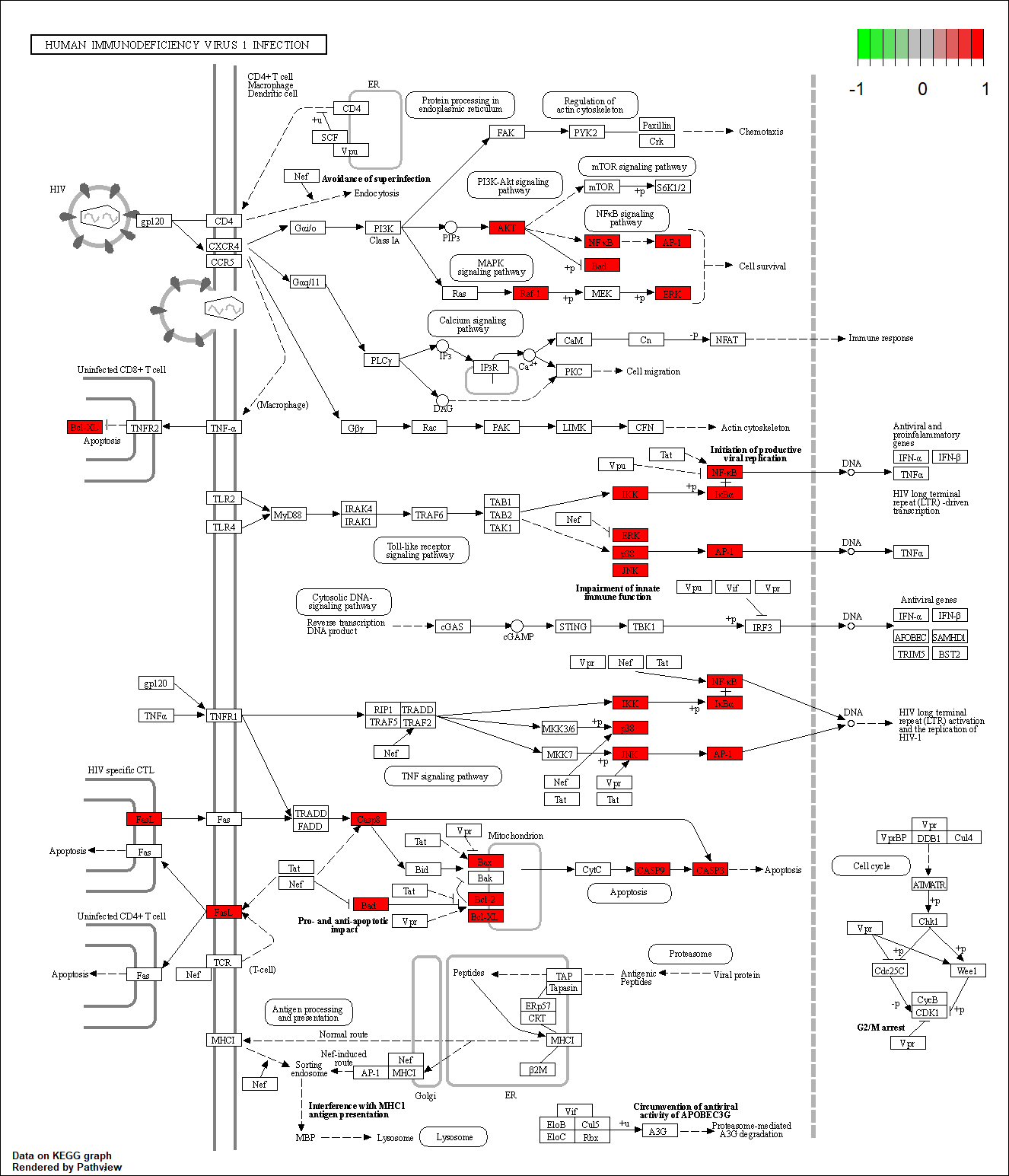


hsa05164. Influenza A


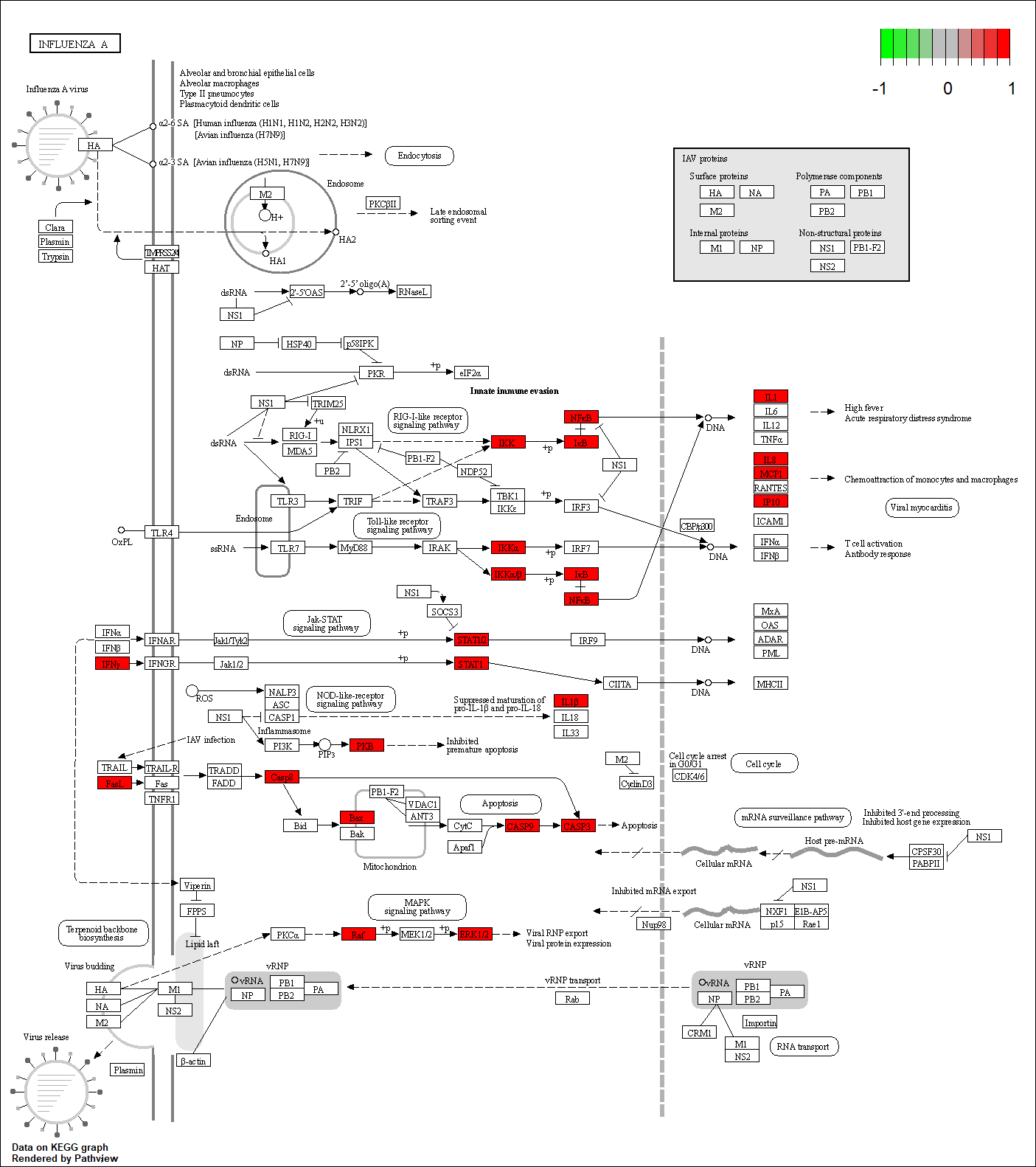


hsa05169. Epstein-Barr virus infection


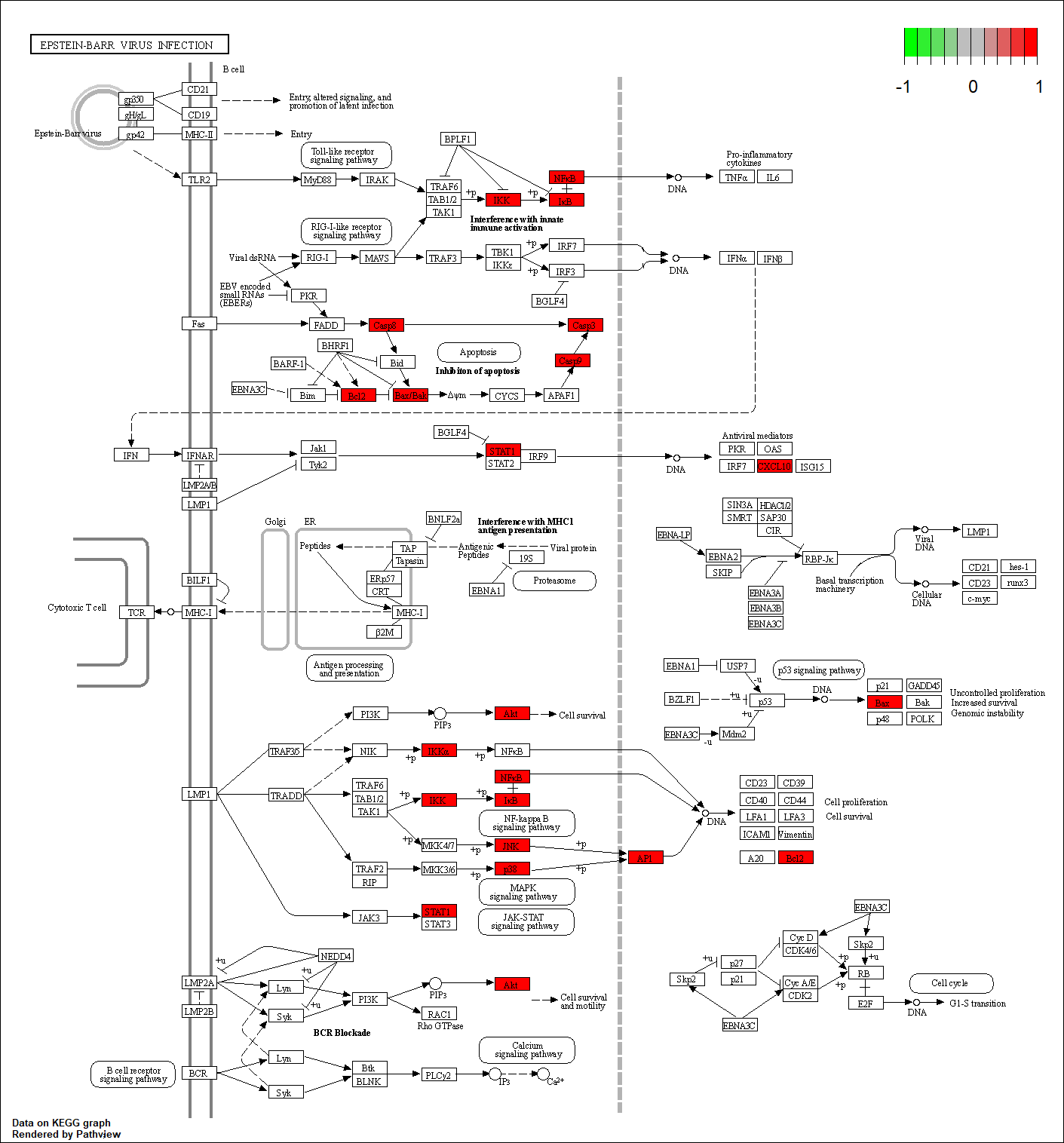


hsa05165. Human papillomavirus infection


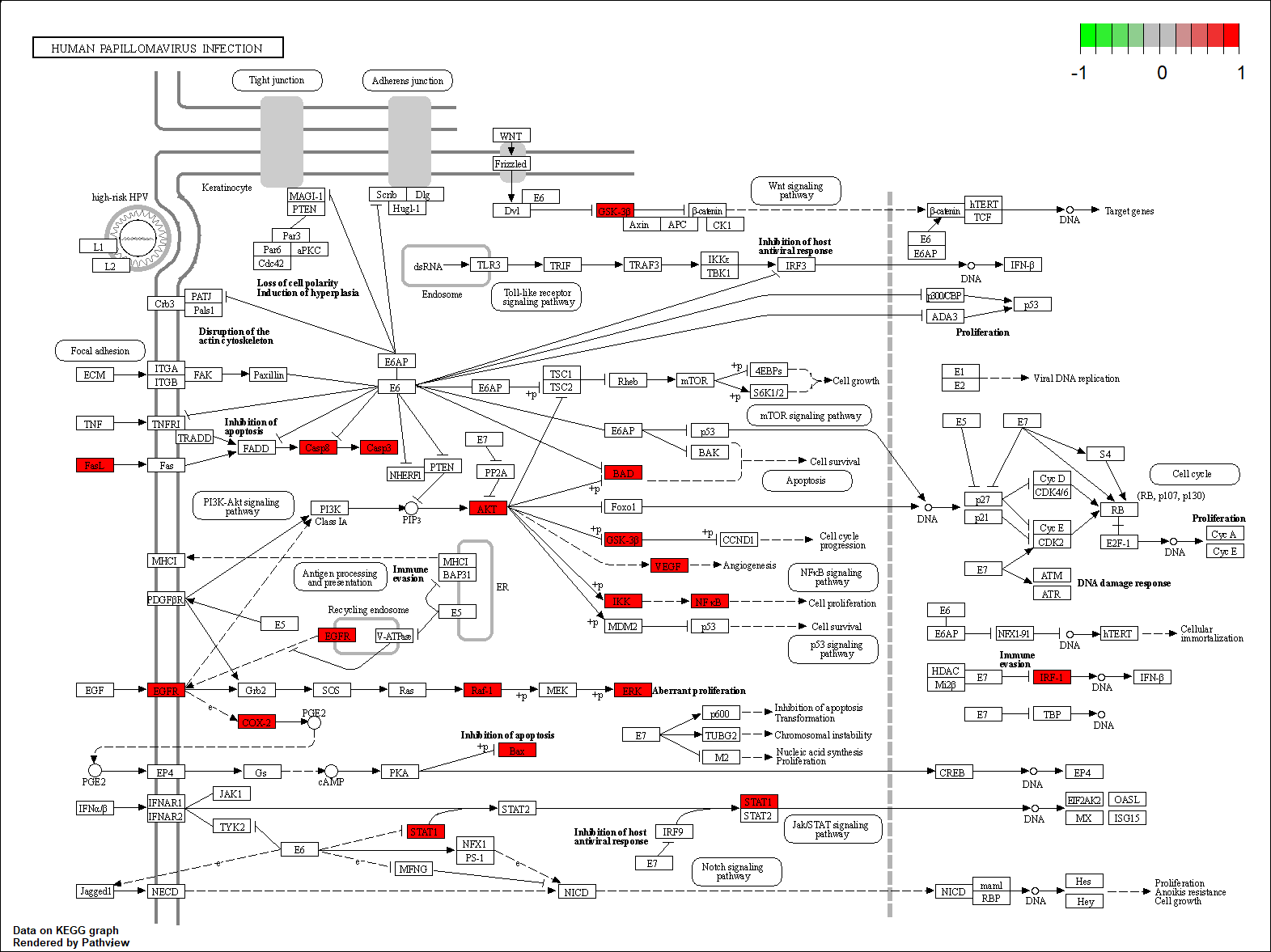


hsa05166. Human T-cell leukemia virus 1 infection


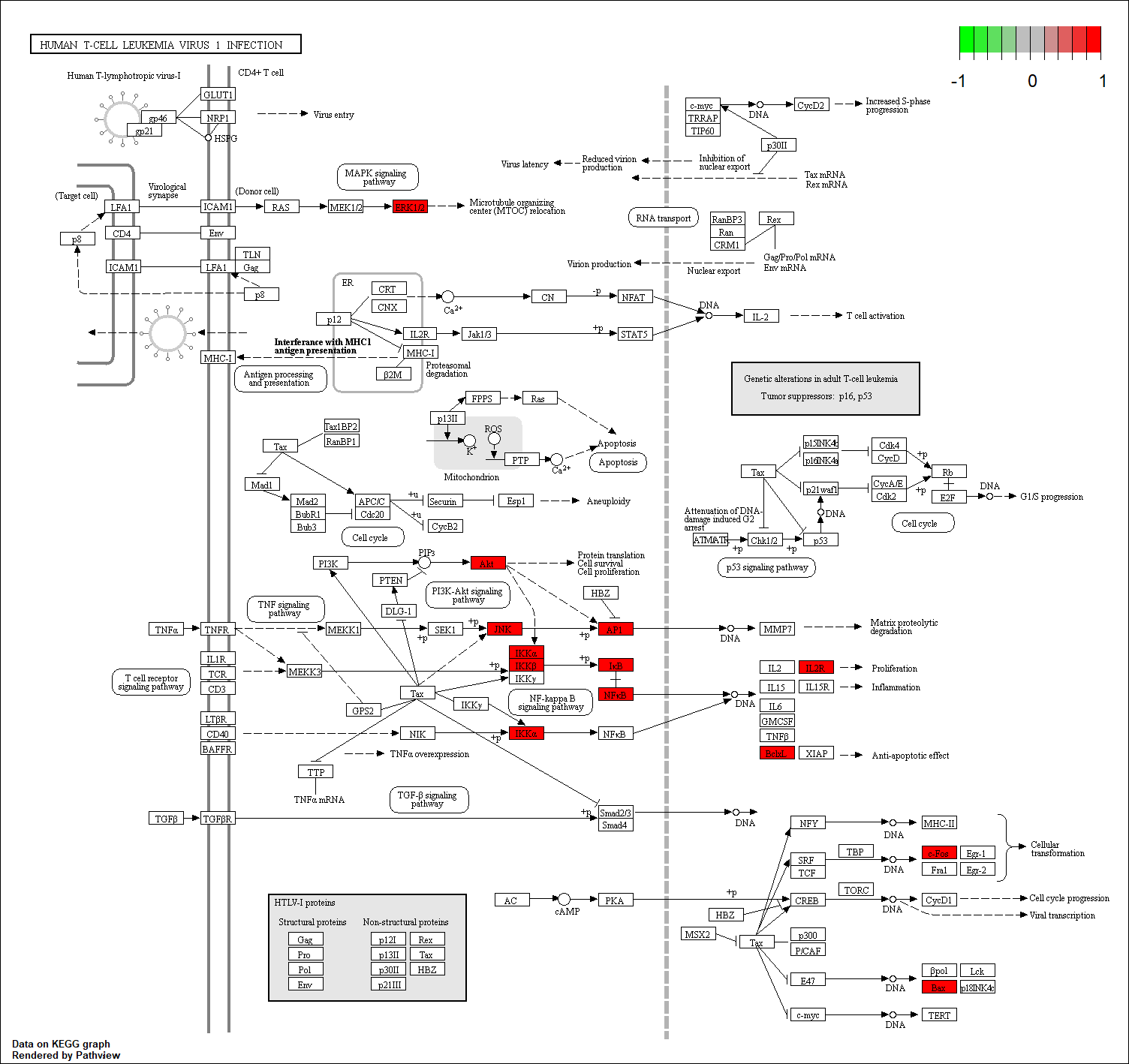


hsa05203. Viral carcinogenesis


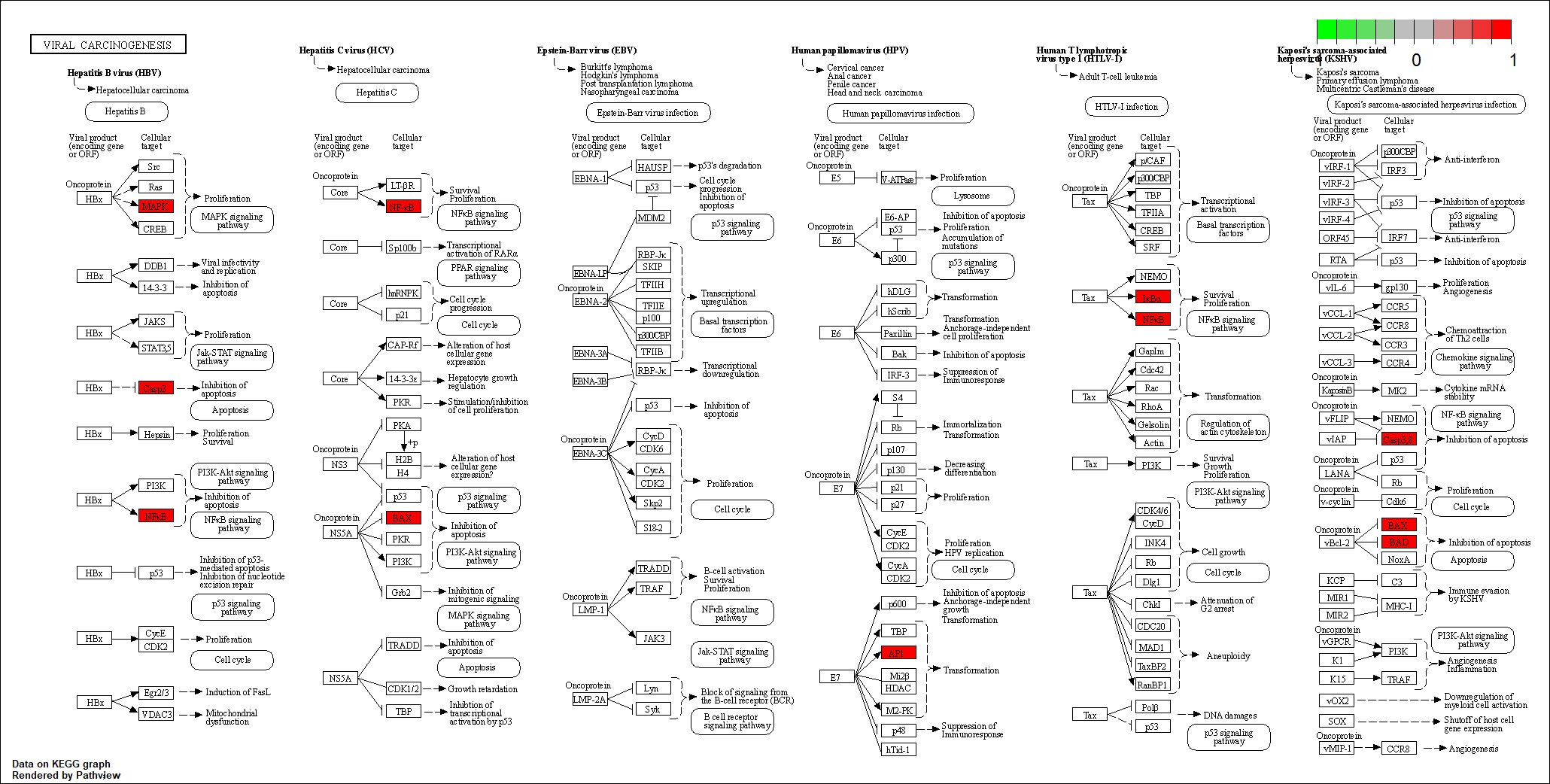


hsa04061. Viral protein interaction with cytokine and cytokine receptor


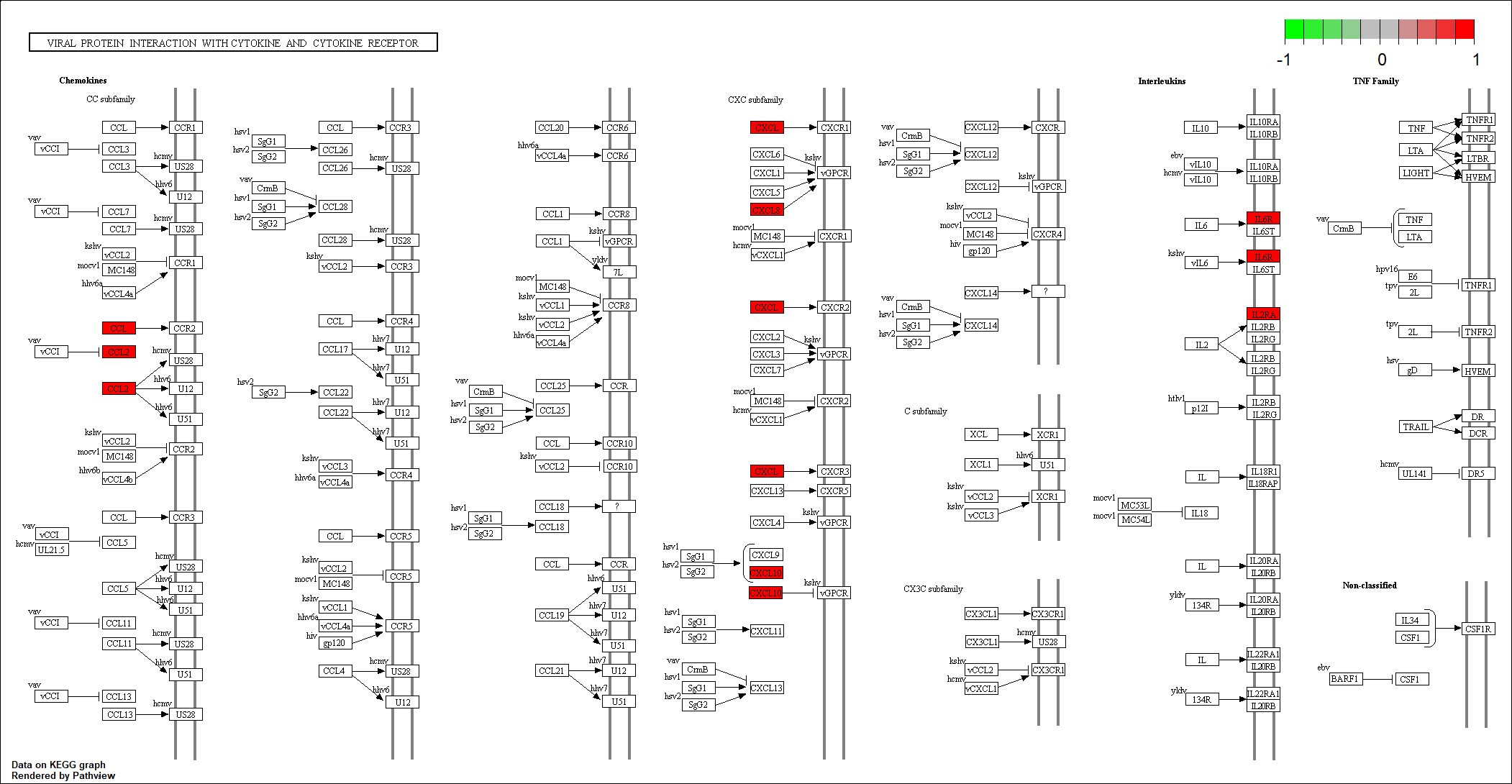


hsa05416. Viral myocarditis


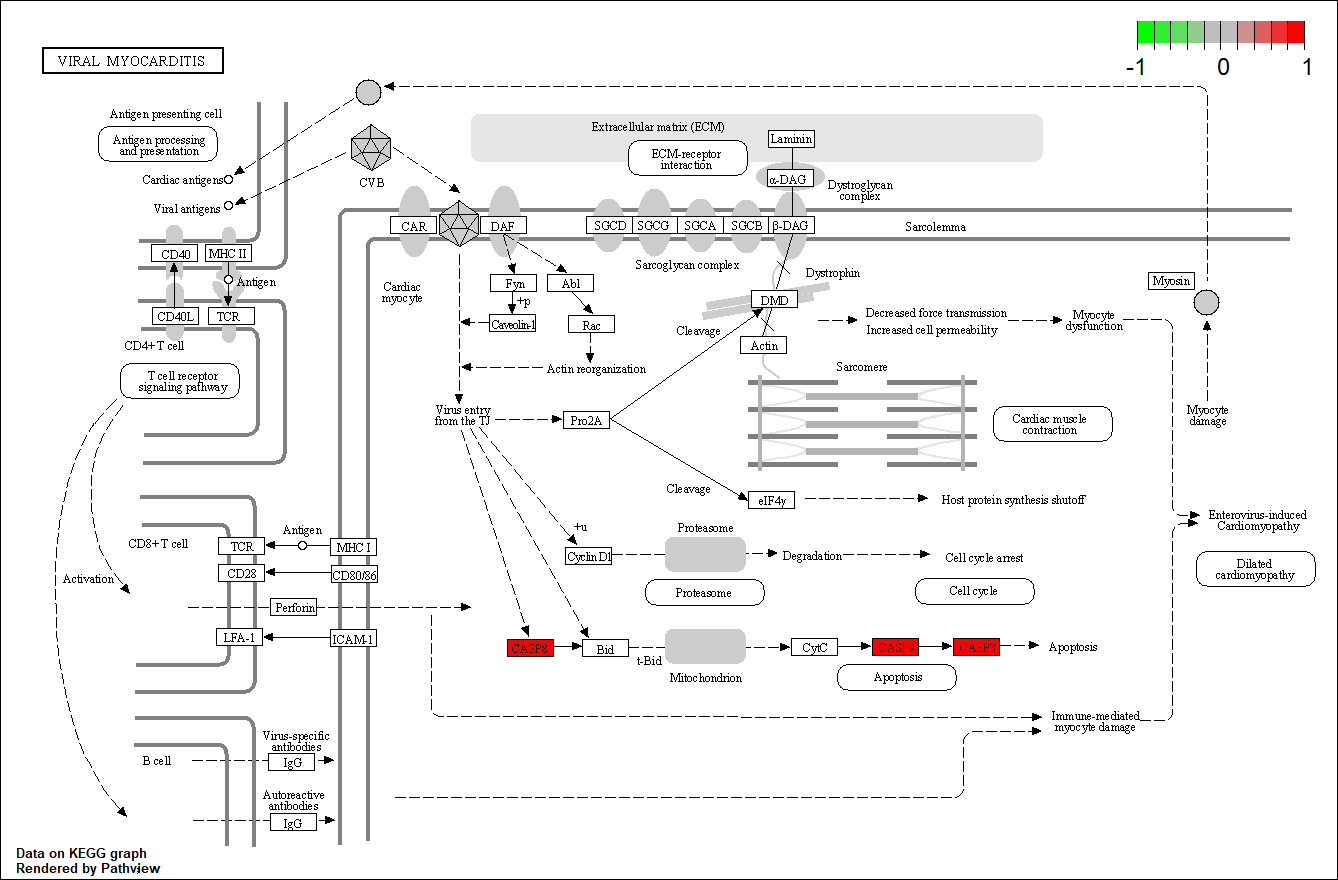

Supplement: Supplementary file 1 — Supplementary Material [file CPR-53-e12949-s001.docx]
